# Bacterial diversity in the intestinal mucosa of heart failure rats treated with Sini Decoction

**DOI:** 10.1186/s12906-022-03575-4

**Published:** 2022-03-30

**Authors:** Zhenyu Zhao, Jiahao Liu, Yanzhi Hu, Xining Zhang, Liqin Cao, Zhenhua Dong, Lin Li, Zhixi Hu

**Affiliations:** 1 Hunan University of Chinese Medicine, Changsha, Hunan China; 2 Institute of Traditional Chinese Medicine Diagnostics, Hunan University of Chinese Medicine, Changsha, Hunan China; 3 The Domestic First-Class Discipline Construction Project of Chinese Medicine, Hunan University of Chinese Medicine, Changsha, Hunan China

**Keywords:** Sini decoction, Metoprolol, Heart failure, Intestinal mucosal bacteria, 16S rRNA gene sequencing

## Abstract

**Background:**

Sini Decoction (SND), a classic Chinese medicine prescription, has been proved to have a good effect on heart failure (HF), whereas its underlying mechanism is still unclear. In order to explore the therapeutic mechanism of SND, we combined with 16S rRNA gene sequencing to analyze the composition of gut microflora in rats with HF.

**Material and methods:**

Twenty Sprague–Dawley (SD) rats were divided into four groups (*n* = 5): normal group, model group, SND treatment group (SNT group), and metoprolol (Met) treatment group (Meto group). All the rats except the normal group were intraperitoneally injected with doxorubicin (concentration 2 mg/mL, dose 0.15 mL/100 g) once a week to induce HF. After successfully modeling, SND and Met were gavaged to rats, respectively. After the treatment period, blood was collected for hematological analyses, myocardial tissue and colon tissues were collected for Hematoxylin–Eosin (H&E) staining, and mucosal scrapings were collected for Illumina Miseq high-throughput sequencing.

**Results:**

Echocardiographic results suggested that both left ventricular ejection fraction (LVEF) and left ventricular fraction shortening (LVFS) in Model rats decreased compared with normal rats. The results of H&E staining showed that compared with the model group, the structures of myocardial tissue and colon tissue in the SNT group and Meto group showed a recovery trend. Alpha results showed that the model group had higher species diversity and richness compared with the normal group. After treatment, the richness and diversity of intestinal bacteria in the SNT group were significantly restored, and Met also showed the effect of adjusting bacterial diversity, but its effect on bacterial richness was not ideal. At the Family level, we found that the number of several bacteria associated with HF in the model group increased significantly. Excitingly, SND and Met had shown positive effects in restoring these HF-associated bacteria. Similarly, the results of Linear discriminant analysis (LDA) showed that both SND and Met could reduce the accumulation of bacteria in the model group caused by HF.

**Conclusion:**

Collectively, SND can improve HF by regulating the intestinal flora. This will provide new ideas for the clinical treatment of patients with HF.

**Supplementary Information:**

The online version contains supplementary material available at 10.1186/s12906-022-03575-4.

## Background

Heart failure (HF), as the end stage of various kinds of cardiovascular diseases (CVDs), is a complex clinical syndrome [1]. According to the statistics, approximately 63 million people suffer from HF worldwide, affecting 1% to 2% of adults and more than 10% of individuals over 70 years of age. In addition, the overall prognosis of patients with HF is lacking, as well as high mortality risk and a high incidence of hospital readmission [2]. Studies have shown that the 6-month readmission rate of patients with HF is 50% [3], and approximately 50% of patients die within 5 years after the initial diagnosis, higher than most types of cancer [4]. As life expectancy increases, the number of patients with HF will grow. In the past few decades, the treatment of HF has been mainly based on western medicine. Although much experience has been accumulated, the effectiveness of the current treatment is still unsatisfactory. Moreover, patients with HF have many cardiovascular comorbidities (atrial fibrillation, hypertension) and non-cardiovascular comorbidities (renal impairment, chronic obstructive pulmonary disease). Obvious side effects may occur when drugs are used to treat HF. For example, diuretics-induced hyponatremia may induce arrhythmias, and beta-blockers may aggravate tachypnea in patients with reversible respiratory diseases [5–7].

Given the present dilemma, it is necessary to seek an "antidote" in the field of traditional Chinese medicine (TCM). Currently, many TCM injections have been used clinically, such as Shenfu injection [8], Shenmai injection [9], Huangqi injection [10], and so forth. These drugs are all derived from classical Chinese prescriptions and have achieved good clinical results. As a famous Chinese prescription, SND comes from the classic book Treatise on Febrile Diseases of the Eastern Han Dynasty [11]. It consists of three Chinese herbal medicines: aconite, dried ginger, and licorice, and has been widely used in the treatment of CVDs for many years with apparent curative effects [12].

The intestinal mucosa is one of the largest immune active organs in the human body and exerts its effects in preventing the invasion of foreign microorganisms [13]. Intestinal mucosa contains about 100 trillion microorganisms called "intestinal microbiota", of which there are more than 15,000 kinds of bacteria [14]. Firmicutes and Bacteroidetes are the most representative microorganisms in intestinal microbiota [15]. Intestinal flora regulates various host functions such as the intestinal barrier, nutrition and immune homeostasis [16]. Once the mucosal barrier and microbial flora are destroyed, the related intestinal diseases will occur, such as CVDs, diabetes, inflammatory bowel disease [15, 17]. Studies suggested that gut microbiota plays an vital role in the occurrence and development of HF. It influences the synthesis and release of several metabolites, including trimethylamine-N-oxide (TMAO), bile acids (BAs) and short chain fatty acids (SCFAs) [18]. A previous study revealed [19] that the decrease of anti-inflammatory symbiotic *F.Prausnitzii* and the increase of pro-inflammatory symbiotic *R.gnavus* in intestinal flora are the essential characteristics of gut microbiota in patients with chronic HF. Kamo T et al. [20] observed that the richness of *Clostridium* and *Dorea* in the gut flora of HF patients is lower than that of healthy subjects at the genus level, and the richness of *Eubacterium rectale* and *Dorea longicatena* at the species level is also notably lower than that of healthy people.

Studies have found that the Chinese herb can affect the occurrence of diseases by regulating intestinal flora [21]. However, several studies have demonstrated that TCMs are often taken orally and show a low oral bioavailability. But after oral administration, the ingredient of TCMs, most of unabsorbed compounds, can easily reach the colon where great majority of intestinal microbes reside. The colon is a comfortable environment where unabsorbed compounds and gut microbiota directly contact and interact with each other. As a result, many components of TCMs are produced with potent bioactivity after the metabolism of gut microbiota and exerting therapeutic effects [22, 23]. For example, zengye decoction can decrease the level of bacteria *Ruminococcus*, *Desulfovibrio*, and *Prevotella* in constipation patients and increase the level of bacteria *Oxalobacter*, *Clostridium* and *Rosa*, which in turn improves the balance of intestinal ecological [24]. Zhao Liping et al. [25] screened 268 kinds of main bacteria from 6082 kinds of bacteria in the intestines of rats on the high-fat diet before and after taking berberine. The results showed that 174 kinds of bacteria are reduced or disappeared (mostly harmful bacteria), 94 kinds of bacteria are increased (mostly beneficial bacteria), and the indexes of endotoxin and inflammation in serum are decreased. It was suggested that extracts of TCM berberine could reduce the host inflammation reaction and improve insulin resistance by regulating intestinal flora. Chang et al. [26] found that Ganoderma lucidum hyphae plays a vital role in treating of coronary heart disease by reducing the proportion of Firmicutes to Bacteroidetes and the number of *Escherichia coli*, increasing the abundance of beneficial bacteria *Clostridium* and *Eubacillus*. Tong et al. [27] used metformin and TCM compound to treat patients with hyperlipidemia and type 2 diabetes, finding that TCM compound improves lipid and glucose homeostasis by increasing the number of co-enrichment bacteria *Blautia spp* and *Coprobacillus*. It can be seen that intestinal flora may be an essential target in treating diseases by TCM.

The efficacy of SND in the treatment of HF has been confirmed by a large number of clinical practices and basic studies [28, 29], but its specific mechanism is uncertain. Therefore, we used 16S rRNA gene sequencing technology to explore the effect of SND on intestinal flora in HF rats.

## Material and methods

### Animals

Twenty male (SD) rats (license No. SCXK (Hunan) 2019–0004), weight ranging from 200 to 220 g, were purchased from Hunan Slake Jingda Experimental Animal Co. Ltd., China. All animals were acclimatized for seven days before starting the experiments.

### Medicine

SND was bought from the First Affiliated Hospital of Hunan University of Chinese Medicine and was identified by a Professor. Basing on the Chinese Pharmacopoeia 2010 edition, SND was formed by Aconite 7.5 g (Chinese herbs pieces, Lot No.: DX200201, Anhui Bozhou Qiancao Traditional Chinese Medicine Decoction Pieces Co. Ltd.); licorice 10 g (Chinese herbs pieces, Lot No.: 2003059, Anhui Bozhou Qiancao Traditional Chinese Medicine Decoction Pieces Co. Ltd.); dried ginger 15 g (Chinese herbs pieces, Lot No.: 2020070602, Hunan Renshang Pharmaceutical Co. Ltd.) [29]. Then, the three herbs were put into a clay pot filled with distilled water and soaked for 24 h at room temperature. The mixture was decocted to the final concentration of 1 g/ml and stored at 4 °C for further use [28].

### Reagents and instruments

Doxorubicin (APExBIO Company, Lot No.: A39662 337,769); Metoprolol tartrate (AstraZeneca Pharmaceutical (China) Co. Ltd., Lot No.: 0902066); 4% paraformaldehyde (Changsha Weisher Biotechnology Co. Ltd., Lot No.: WB03004A); DNA extraction kit (E.Z.N.A.® Soil DNA Kit, Omega Bio-Tek, USA); AxyPrep DNA Gel Extraction Kit (Axygen biosciences, axygen, USA); MiSeq PE300 (Illumina, USA); Magnetic frame (China Bioengineering (Shanghai) Co. Ltd); Sequencer (Illumina Miseq, Illumina, USA); PCR instrument (ABI GeneAmp® 9700, ABI, USA); Electrophoresis instrument (DYY-6C, Beijing Liuyi Instrument Factory, China); Database building kit (NEXTFLEX® Rapid DNA-Seq Kit, Bioo Scientific, USA); Agarose (biowest Agarose, biowest, Spain); FastPfu Polymerase (FastPfu Polymerase, TransGen, China); Sequencing kit (MiSeq Reagent Kit v3, Illumina, USA).

### Modeling and treatment

Both doxorubicin solution and Met solution were prepared with saline. After adaptive feeding for seven days, all rats were classified into the normal group (5 rats) and HF-induced group (15 rats) according to random number table. The normal group was intraperitoneally injected with saline once a week, while the HF-induced group was intraperitoneally injected with doxorubicin once a week at a concentration of 2 mg/ml and a dose of 0.15 mL/100 g [30]. After 7 weeks, the model of HF was confirmed by echocardiography. Then, the fifteen rats in the HF-induced group were randomly assigned to the model group, Sini Decoction treatment group (SNT group) and metoprolol treatment group (Meto group), with 5 rats in every group. The Meto group (metoprolol, concentration 2 mg/mL, dose 10 mg/kg) and SNT group (Sini Decoction, concentration 1 g/mL, dose 2.8 g/kg [29]) were given the corresponding drugs by gavage for 21 days [12], and the normal and model group were fed the same volume of saline via intragastric administration.

### Echocardiography

Echocardiography was performed in all rats using SonoScape-S2N ultrasound system. LVEF and LVFS were calculated by the four-point method to assess left ventricular systolic function [31].

### Sample collection

After the end of the experiment, all rats were anesthetized with pentobarbital sodium. Blood samples were taken from the abdominal aorta and centrifuged once at 4℃, 3000 RPM for 15 min. The upper serum was obtained and stored in a -80℃ refrigerator for subsequent analysis. Myocardial tissue were extracted and stored in 4% paraformaldehyde. The colon segment of rats was cut on an aseptic operating platform. Then, colonic mucosa was scraped with coverslips to squeeze out the chymus, and the intestinal tract was cut open. The intestinal wall was cleaned with saline, and then stored in a cryopreservation tube. Finally, the intestinal mucosal samples were immediately immersed in liquid nitrogen for 1 min and stored at 4℃ for the following experiment.

### DNA extraction

According to the instructions of E.Z.N.A.® soil DNA kit, the total DNA of the microbial community was extracted from the collected intestinal mucosa samples. The extraction quality of DNA was detected by 1% agarose gel electrophoresis, and the concentration and purity of DNA were detected by NanoDrop2000.

### PCR amplification and Illumina MiSeq sequencing

The hypervariable region V3-V4 of the bacterial 16S rRNA gene were amplified with primer pairs 338F (5'-ACTCCTACGGGAGGCAGCAG-3') and 806R (5'-GGACTACHVGGGTWTCTAAT-3') by an ABI GeneAmp® 9700 PCR thermocycler. The amplification procedure was as follows: ① Pre-denaturation, 95℃, 3 min. ②27 cycles (denaturation at 95℃ for 30 s, annealing at 55℃ for 30 s and extension at 72℃ for 30 s)③ Stable extension at 72℃ for 10 min. ④ preservation, 4℃. The PCR mixtures contain 4 μL of 5 × TransStart FastPfu buffer, 2.5 mM dNTPs 2 μL, forward primer (5 μM) 0.8 μL, reverse primer (5 μM) 0.8 μL, TransStart FastPfu DNA Polymerase 0.4 μL, template DNA 10 ng, and finally ddH2O up to 20 μL. The PCR reactions were carried out in triplicate. The PCR product was extracted from 2% agarose gel and purified using the AxyPrep DNA Gel Extraction Kit according to manufacturer’s instructions and quantified using Quantus™ Fluorometer.

In the light of standard protocols of Majorbio Bio-Pharm Technology Co. Ltd. (Shanghai, China), the purified amplicons were pooled in equimolar and paired-end sequenced on an Illumina MiSeq PE300 platform (Illumina, San Diego, USA).

### Processing of sequencing data

The raw 16S rRNA gene sequencing reads were demultiplexed, quality-filtered by fastp (https://github.com/OpenGene/fastp, version 0.20.0) and merged by FLASH (http://www.cbcb.umd.edu/software/flash, version 1.2.7). Operational taxonomic units (OTUs) with 97% similarity cutoff were clustered using UPARSE (http://drive5.com/uparse/, version 7.1), and chimeric sequences were recognized and removed. The taxonomy of each OTU representative sequence was analyzed by RDP Classifier (http://rdp.cme.msu.edu/, version 2.2) against the 16S rRNA database (SILVA v132) using confidence threshold of 0.7.

### Bioinformatics and statistical analysis

Intestinal mucosa bacterial diversity indices (including ACE, Chao, Shannon, and Simpson) were measured by Mothur (version 1.30.2, https://www.mothur.org/wiki/Download_mothur). Bar and PCoA were conducted with the R package (http://www.R-project.org/). LDA was made by LEfSe (http://huttenhower.sph.harvard.edu/galaxy/root?tool_id=lefse_upload). GraphPad Prism software (version 8.0.2) was used for statistical analysis. *P* < 0.05 or *P* < 0.01 were applied to compare the statistical significance of differences.

## Results

### Graphical abstract of the experiment

In order to help others understand our experimental process. An additional figure file shows this in more detail (see Additional file 1).

### Results of echocardiography and myocardial morphology

**Fig. 1:**
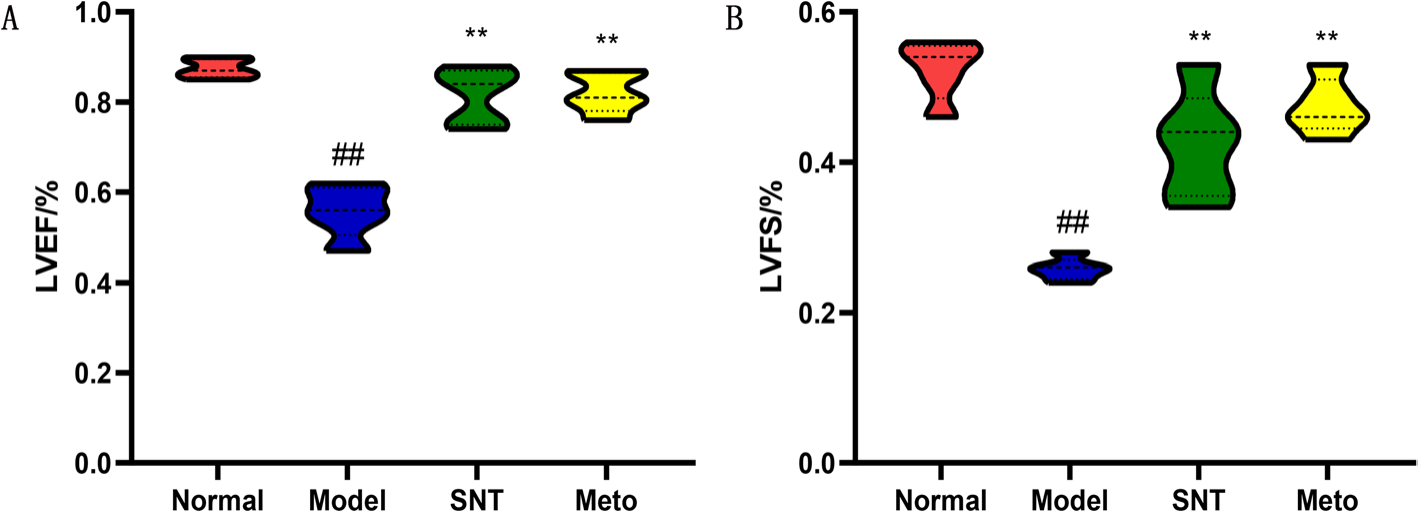
Cardiac function data on LVEF (**A**) and LVFS (**B**). ##*P* < 0.01 versus the normal group; **P < 0.01 versus the model group. Normal, Model, SNT and Meto represent normal group, model group, Sini Decoction treatment group and metoprolol treatment group, respectively

**Fig. 2:**
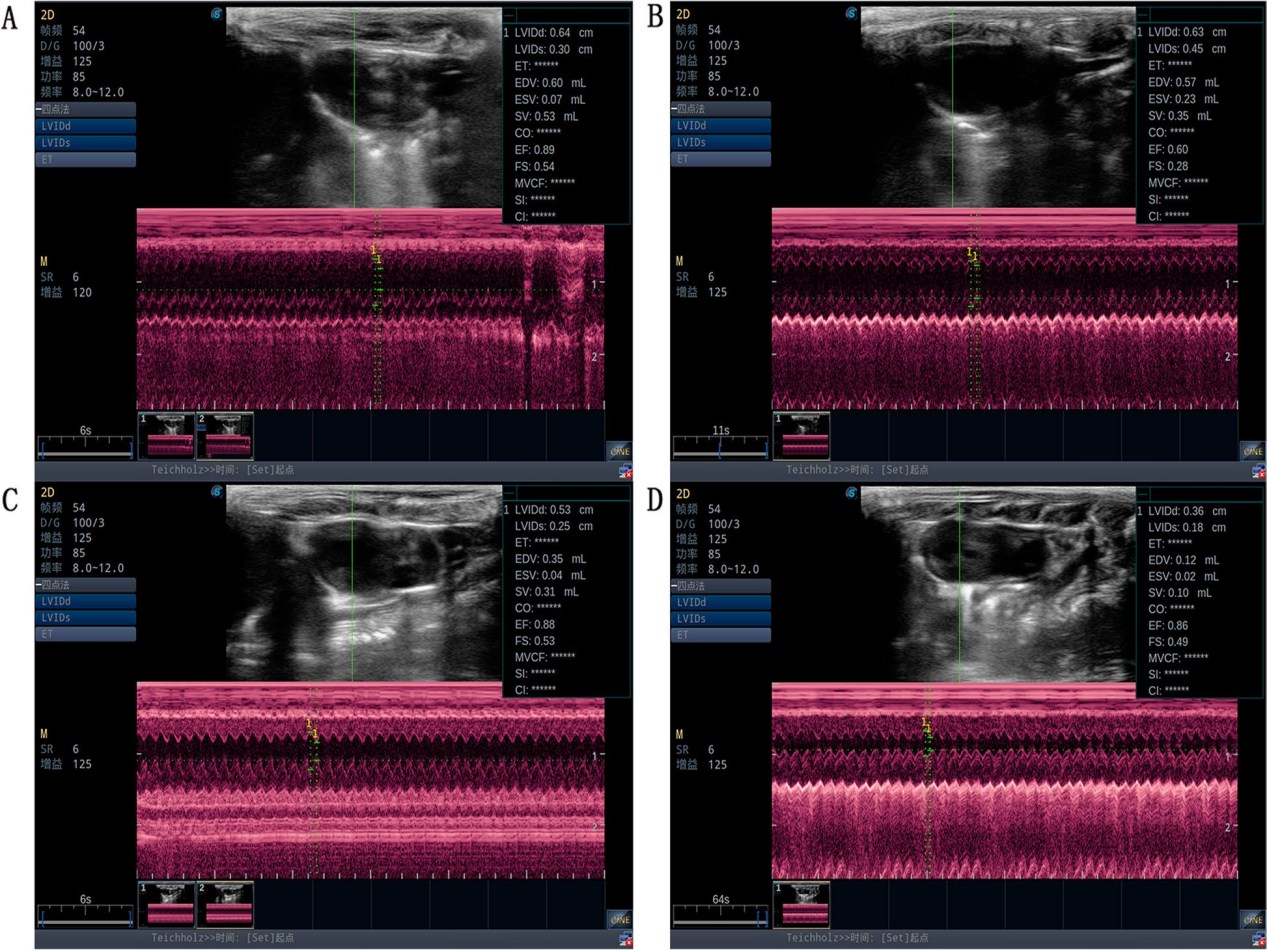
Representative echocardiogram. **A**-**D** represent normal group, model group, Sini Decoction treatment group and metoprolol treatment group, respectively

Compared with the normal group, LVEF and LVFS in the model group decreased significantly (*P* < 0.01), indicating that HF leads to impaired heart function, consistent with existing literature [32]. After treatment, LVEF and LVFS in the SNT and Meto group increased significantly compared with model group (*P* < 0.01) (Figs. 1A, B and 2A-D).

**Fig. 3:**
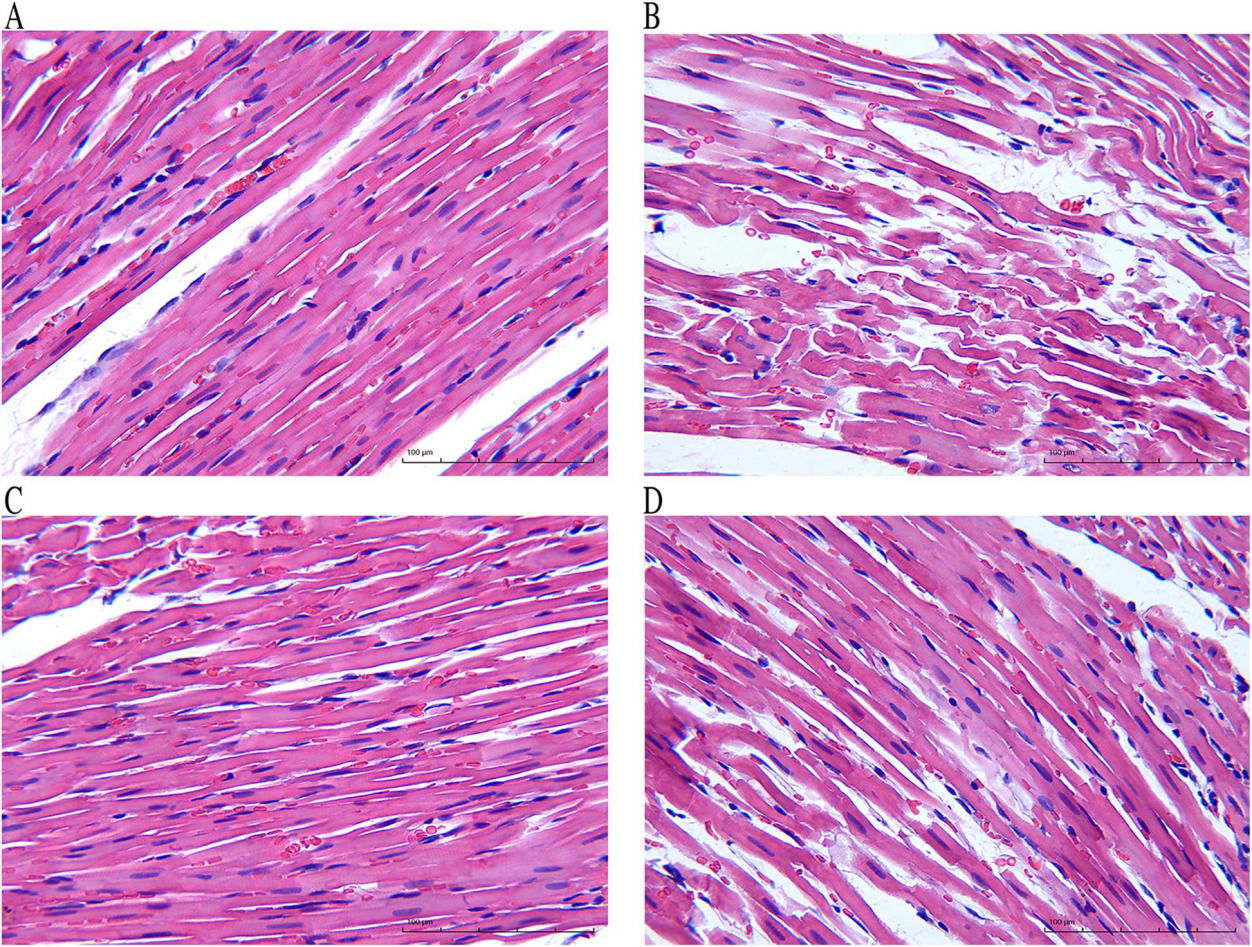
Representative H&E-stained images of Myocardial tissue (× 200, 100 μm). **A-D** represent normal group, model group, Sini Decoction treatment group and metoprolol treatment group, respectively

The results of H&E staining showed that the myocardial structure of the normal group was intact, the myocardial fibers were arranged neatly without breaking, and the nuclei were clear. Myocardial fibers in the model group were disorganized and broken, and myocardial cell necrosis was observed. Compared with model group, myocardial cell destruction in the SNT group was significantly reduced, myocardial fiber was not broken, and myocardial cell necrosis was reduced. The myocardial structure of the Meto group was also restored, but the effect was not as obvious as the SNT group (Fig. 3).

### Influences of Sini Decoction on the histomorphology of intestinal wall

**Fig. 4:**
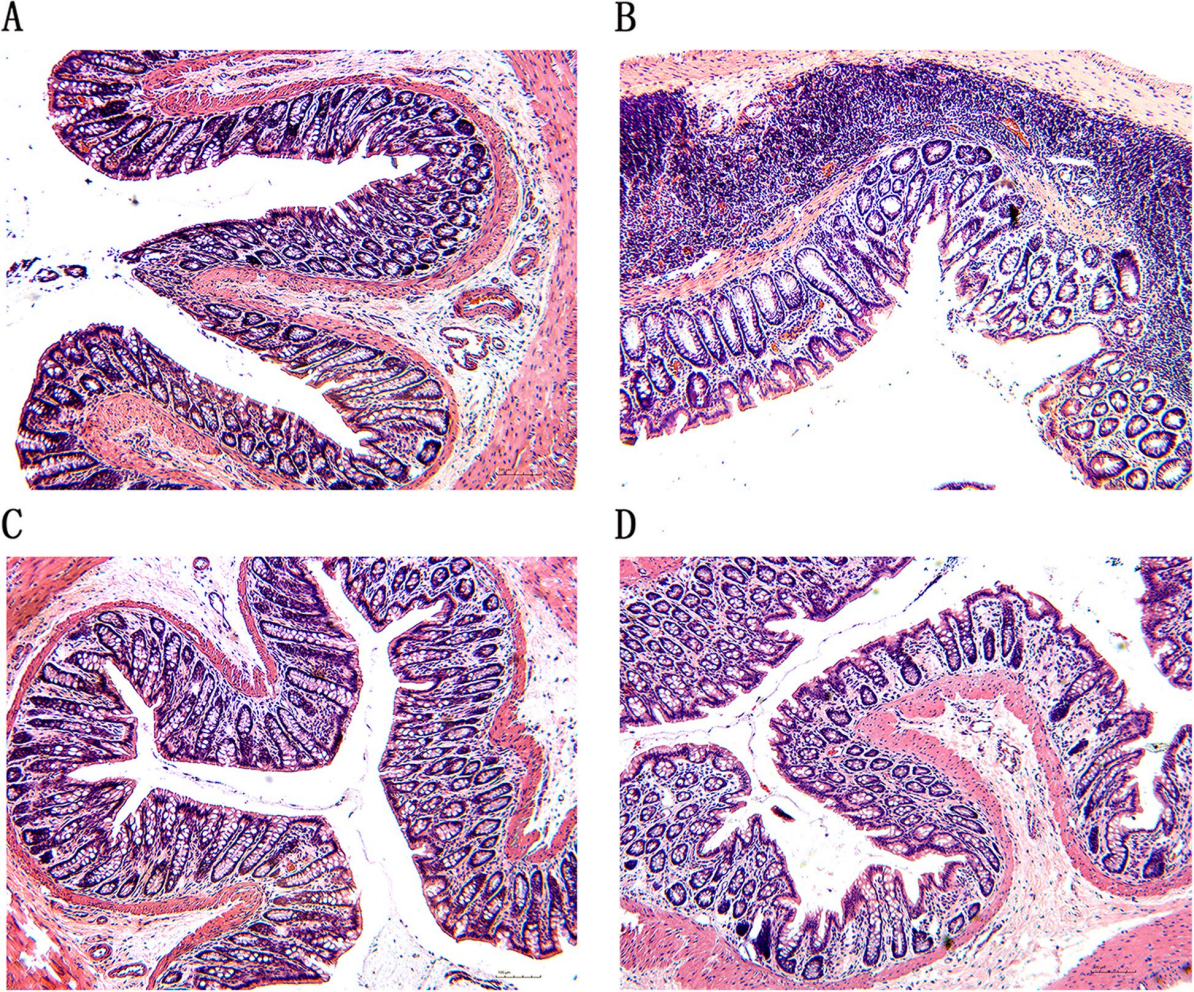
Representative H&E-stained images of colon sections (× 100, 100 μm). **A-D** represent normal group, model group, Sini Decoction treatment group and metoprolol treatment group, respectively

The results were shown in Fig. 4A-D. Results of H&E staining showed that the structure of intestinal wall of the normal group was intact, the chorion was arranged regularly, and there were a small amount of erythrocytes cells and inflammatory cells in the mucosa and submucosa. But the shape of intestinal villi was irregular, the structure of the mucosa layer was destroyed, and Inflammatory cells thoroughly infiltrated the submucosa in the model group. After treatment, the erythrocyte cells and inflammatory cells in the submucosa of the SNT and Meto groups were significantly reduced. And the structure of the intestinal wall in the SNT group had been restored, but Met was not effective in restoring the structure of the intestinal mucosa.

### Effect of Sini Decoction on N-terminal pro-brain natriuretic peptide (NT-proBNP) and endotoxin lipopolysaccharide (LPS)

**Fig. 5:**
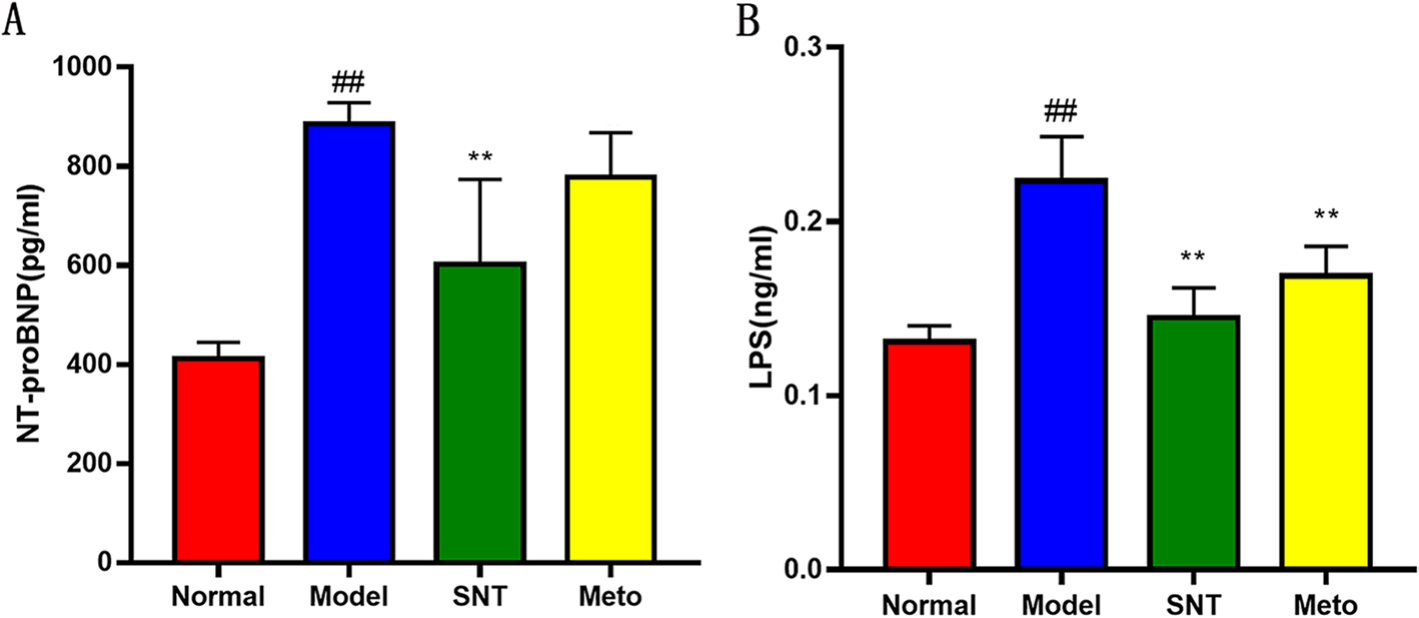
**A** Content of NT-proBNP in serum of rats. **B** Contents of endotoxin lipopolysaccharide in serum of rats. ##*P* < 0.01 versus the normal group; **P < 0.01 versus the model group. Normal, Model, SNT and Meto represent normal group, model group, Sini Decoction treatment group and metoprolol treatment group, respectively

Compared with the normal group, the serum NT-proBNP content of the model group was significantly increased (*P* < 0.01). SND could significantly reduce the serum NT-proBNP content in rats (*P* < 0.01). Met was also beneficial in regulating the serum NT-proBNP content, but, the difference was not statistically significant compared with the model group (*P* > 0.05) (Fig. 5A).

The serum endotoxin LPS content of rats in every group was detected. The content of serum endotoxin LPS in the model group was significantly increased (*P* < 0.01) in comparison with the normal group. After treatment, the level of endotoxin LPS in the SNT group decreased compared with the model group (*P* < 0.01). The Meto group was also significantly decreased (*P* < 0.01), and the correction degree of endotoxin LPS content in the SNT group was better than that of the Meto group (Fig. 5B).

These results suggested that both SND and Met can reduce the serum NT-proBNP content and endotoxin LPS content in HF rats. And SND has a better effect than Met.

### Effect of Sini Decoction on bacterial diversity of intract mucosa in rats with heart failure

**Fig. 6:**
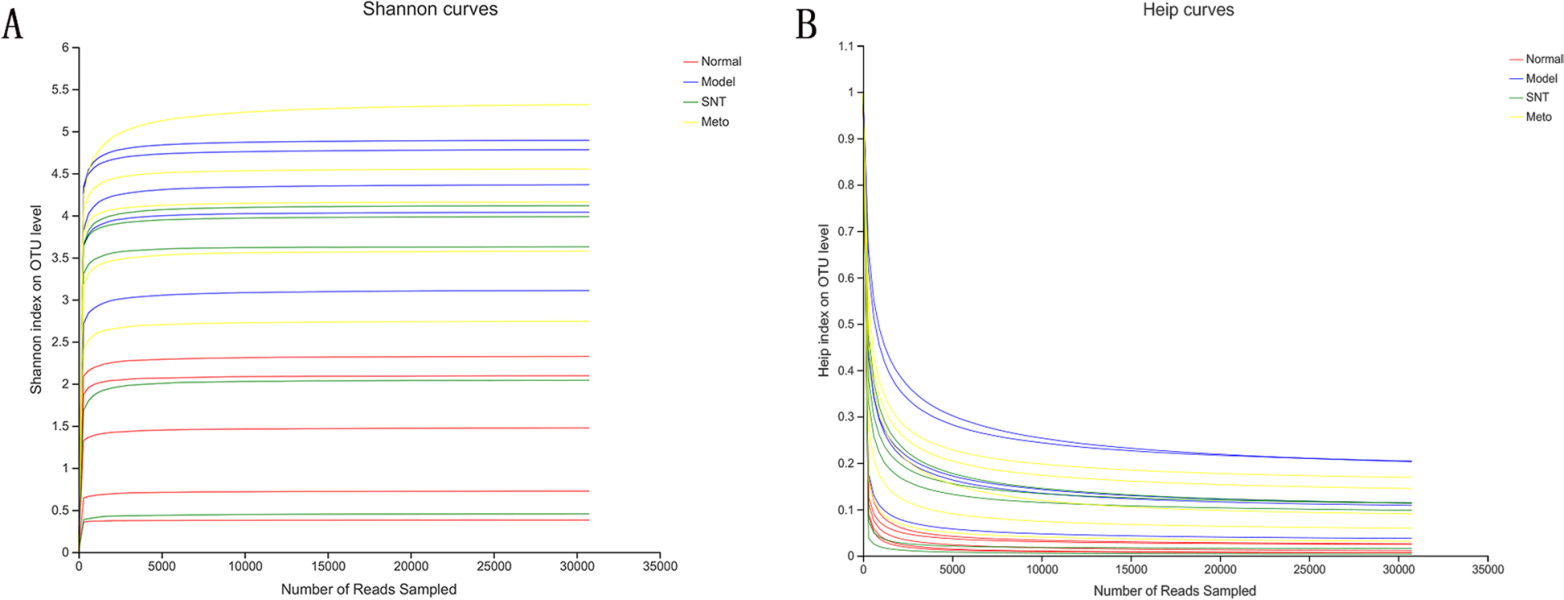
Rarefaction curve. (**A**) Shannon index (**B**) Heip index. As the sequencing quantity increased, the number of species found in each sample tended to be flat. Normal, Model, SNT and Meto represent normal group, model group, Sini Decoction treatment group and metoprolol treatment group, respectively

A total of 617,160 effective sequences were obtained from 20 samples through sequencing and quality control filtering average of 30,858 effective sequences were generated from a single sample. Rarefaction curves tended to flatten with the increase of sequencing volume and extends to the right end of the forehead of the X-axis, indicating that the current sequencing volume was sufficient to be used for subsequent analysis (Fig. 6A, B).

**Fig. 7:**
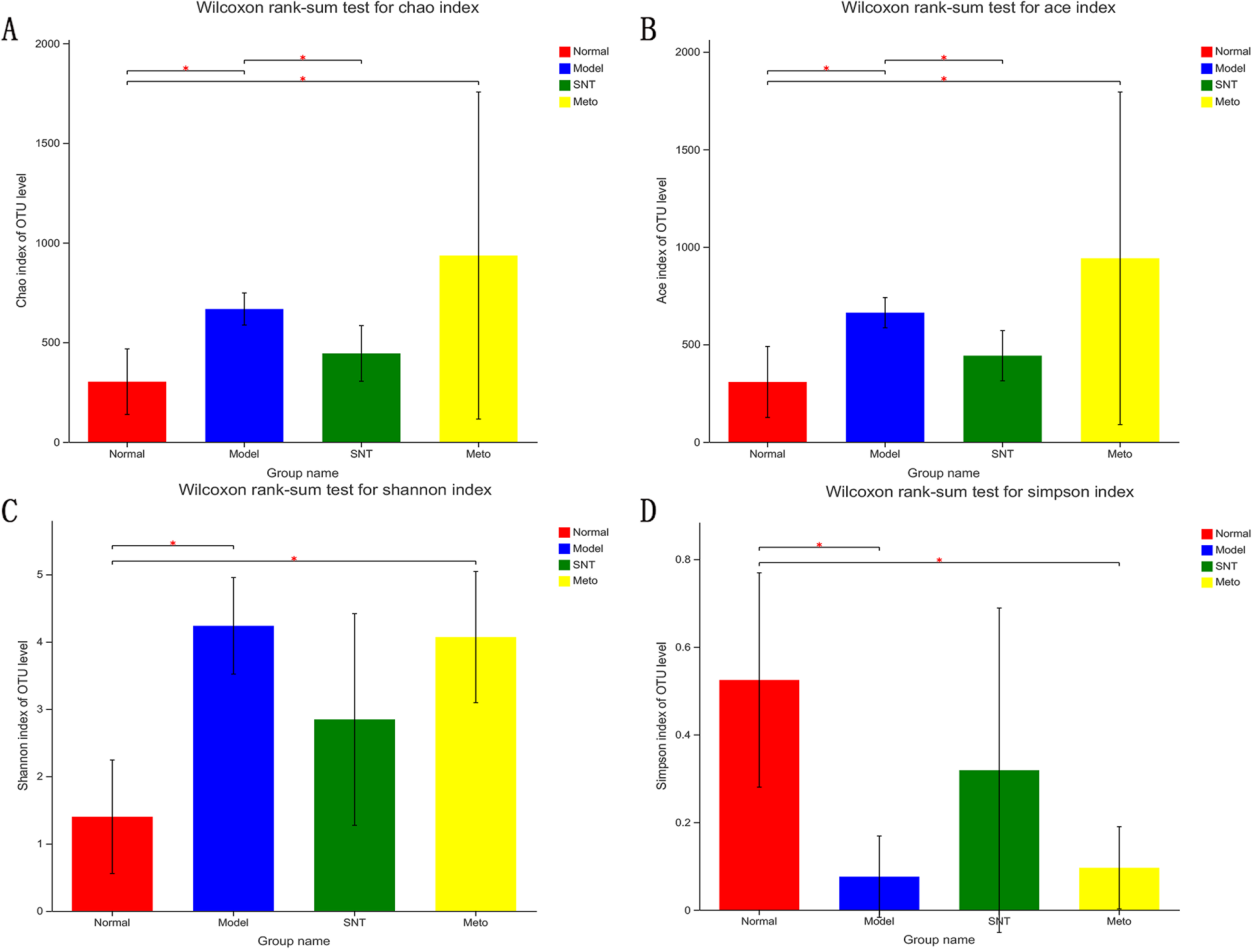
Alpha diversity index. **A**-**D** represent the Chao estimator, the ACE estimator, the Shannon diversity index, and the Simpson diversity index, respectively. *P < 0.05. Normal, Model, SNT and Meto represent normal group, model group, Sini Decoction treatment group and metoprolol treatment group, respectively

A total of 3701 operational taxonomic units (OTUs) were obtained from 20 samples by paired-end sequencing. The quantity of OTUs in the normal, model, SNT and Meto groups were 843, 1340, 1170 and 2856, respectively. This indicated an increase in the quantity of OTUs in HF rats. After processing with SND, the number of OTUs was reduced. But the quantity of OTUs in the Meto group increased further. Alpha diversity analysis is a commonly used indicator to estimate the richness and diversity of microbial species (Fig. 7). Chao and ACE are usually used to calculate species richness; the higher the score, the higher the richness. Shannon and Simpson are commonly used to estimate species diversity. The larger the Shannon index and the smaller the Simpson index, the higher the species diversity is. In this experiment, the values of Chao (*P* < 0.05), ACE (*P* < 0.05) and Shannon (*P* < 0.05) in the model group were significantly higher than those in the normal group, and the value of Simpson (*P* < 0.05) was significantly lower than that in the normal group. After treatment, the values of Chao, ACE, Shannon and Simpson had a callback in the SNT and Meto group. The values of Chao (*P* < 0.05) and ACE (*P* < 0.05) were significantly decreased in comparing with the model group after treatment with SND. The values of Shannon and Simpson in the SNT and Meto group both showed a trend of correction, but they were not statistically significant compared with the model group. Interestingly, the Chao (*P* < 0.05), ACE (*P* < 0.05), Shannon (*P* < 0.05) and Simpson (*P* < 0.05) values of the Meto group were significantly different from the normal group. The specific values of Alpha diversity analysis are shown in Additional file 2.

The results indicated that SND benefits to restore the bacterial richness and diversity of intestinal mucosa of HF rats. Met has an insignificant effect of restoring the diversity of the intestinal mucosal flora, and the effect of restoring the richness of the flora is not ideal.

### Effects of Sini Decoction on the bacterial structure in the intestinal mucosa of rats with heart failure

**Fig. 8:**
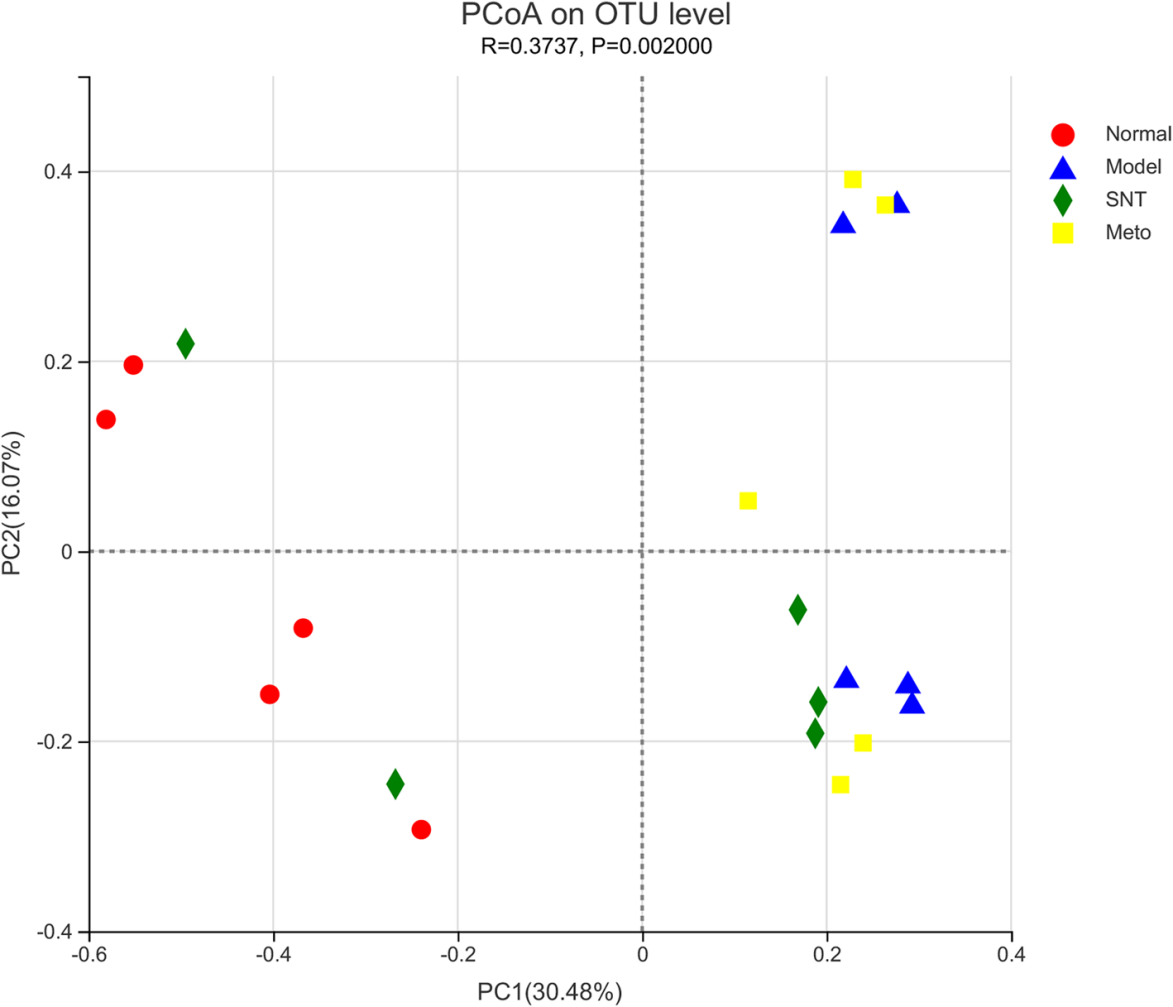
Significant differential principal coordinates analysis (PCoA) based on bray–curtis distance among groups. Each point represents a sample, and points of different colors belong to different samples. The closer the distance between the two points, the higher the similarity of the microbial community structure between the two samples. Normal (Red circle), Model (Blue triangle), SNT (Green diamond), and Meto (Yellow square) represent normal group, model group, Sini Decoction treatment group and metoprolol treatment group, respectively

**Fig. 9:**
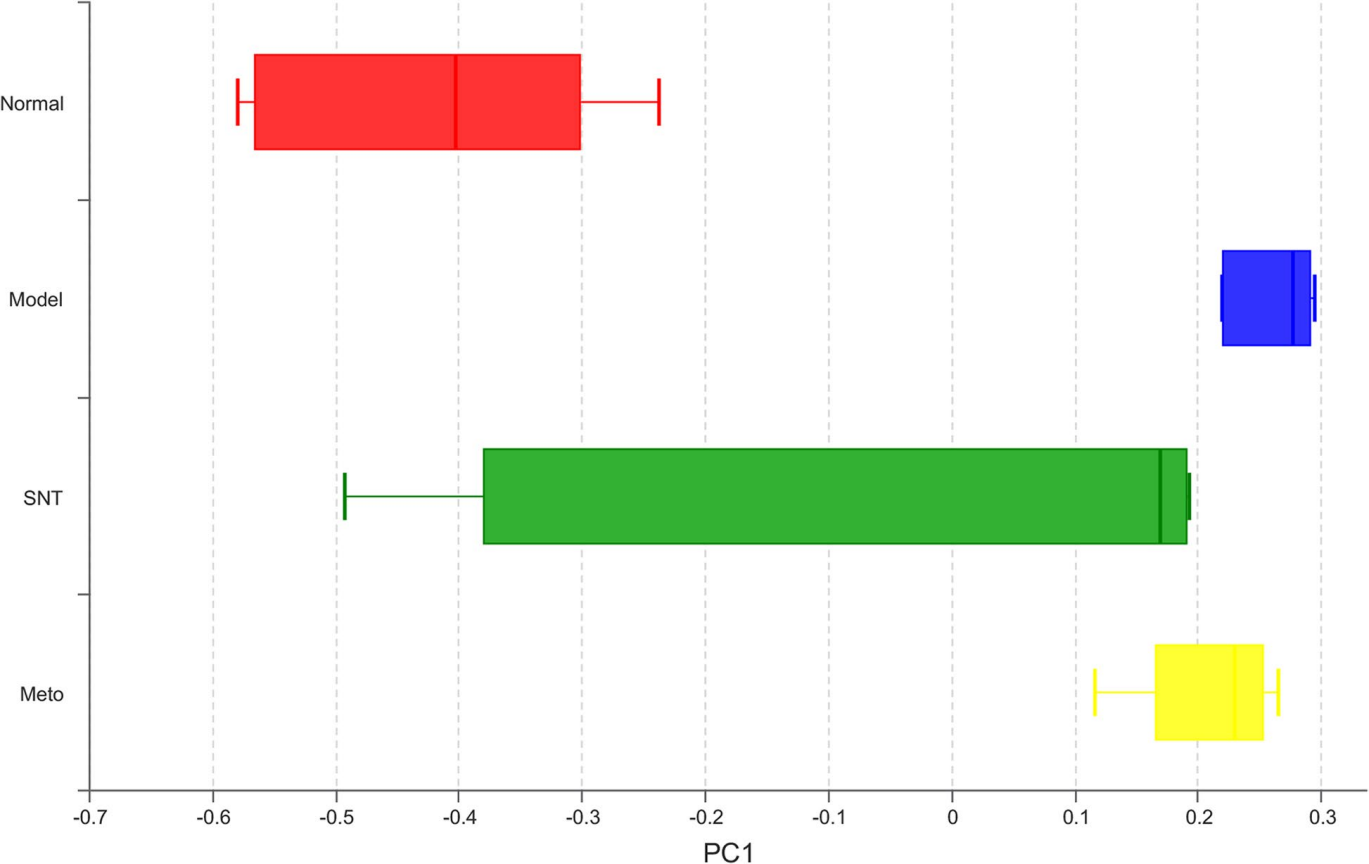
The box plot represents the distribution of different groups of samples on the PC1 axis. Normal (Red box), Model (Blue box), SNT (Green box) and Meto (Yellow box) represent normal group, model group, Sini Decoction treatment group and metoprolol treatment group, respectively

The β-diversity measures changes in the diversity of a species from one environment to another. Principal coordinate analysis (PCoA) based on Bray_Curtis distance algorithm was used to explore the differences of intestinal bacteria in four groups of rats. The representativeness of PC1 and PC2 to the total flora was 30.48% and 16.07% separately. The distance between the samples in the normal and model group was large, indicating that the bacterial structure of the intestinal mucosa changed significantly after HF. After treatment, the distance between the Meto and model group was relatively close, indicating that the effect of Met on the restoration of bacterial structure in the intestinal mucosa of HF rats was not significant. The distance between the SNT and model group became longer and the distance between the SNT and normal group became closer on the PC1 axis. These findings suggested that SND can restore the bacterial structure in the intestinal mucosa of HF rats and its effect was better than that of Met (Figs. 8 and 9).

### Effects on the composition of intestinal mucosal microflora at the phylum level

**Fig. 10:**
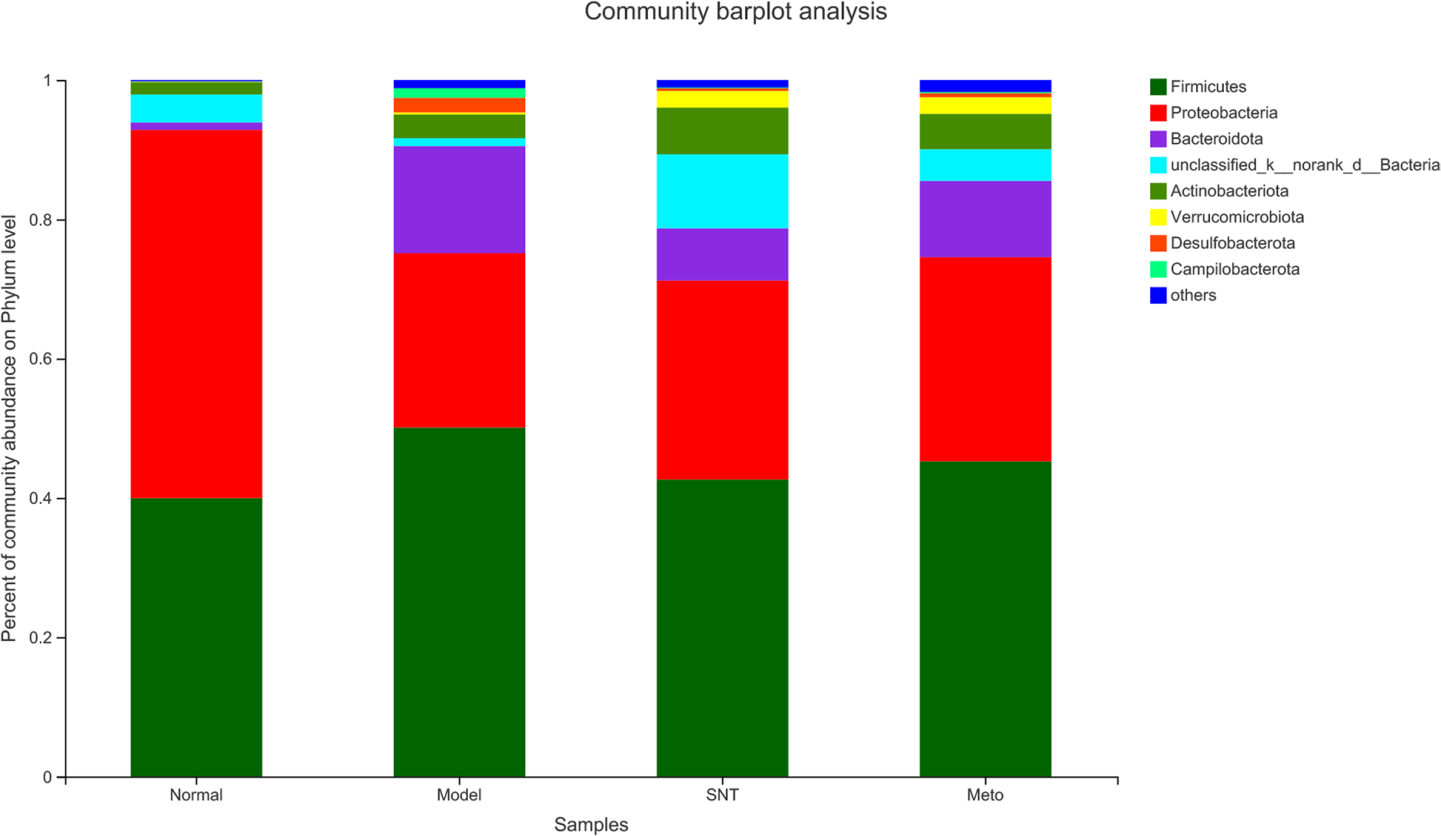
Bacterial community abundance bar plot at the phylum level. Normal, Model, SNT and Meto represent normal group, model group, Sini Decoction treatment group and metoprolol treatment group, respectively

**Fig. 11:**
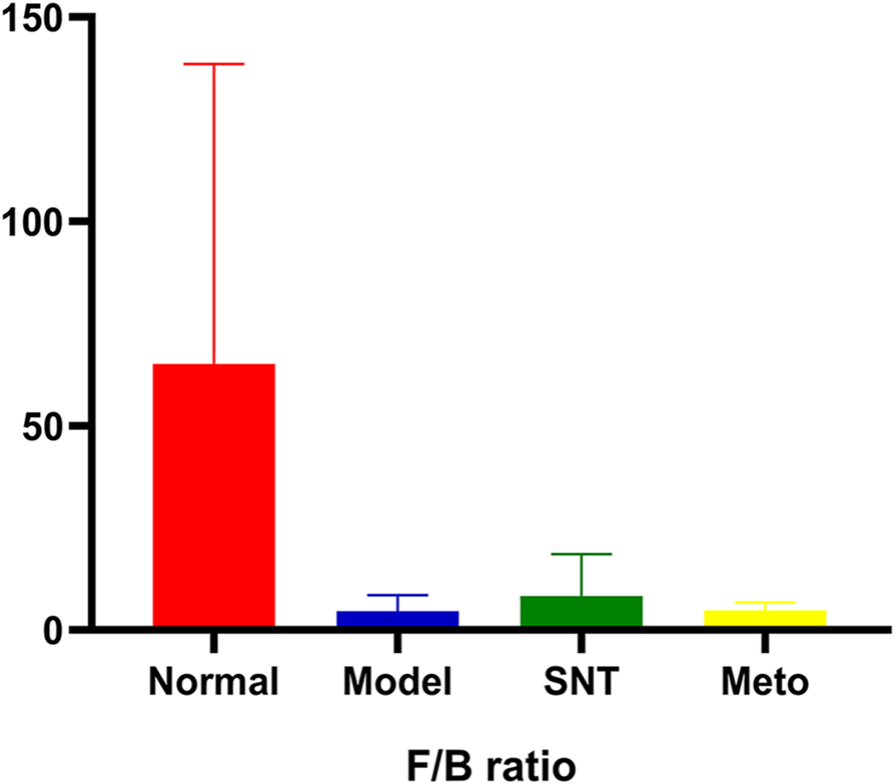
The ratio of Firmicutes/Bacteroidota (F/B ratio). Normal, Model, SNT and Meto represent normal group, model group, Sini Decoction treatment group and metoprolol treatment group, respectively

The percentage bar chart of intestinal mucosa bacterial community reveals the species and relative abundance of bacteria, as shown in Fig. 10. The eight most abundant bacteria were detected at the phylum level and the relative abundance was over 1%. The eight main bacterial phyla of the four groups were Firmicutes, Proteobacteria, Bacteroidota, Unclassified_k__norank_d__Bacteria, Actinobacteria, Verrucomicrobiota, Desulfobacterota, Campilobacterota. The relative abundance of Firmicutes was model group > Meto group > SNT group > normal group, accounting for 50.11%, 45.25%, 42.65% and 39.97% respectively. The relative abundance of Bacteroidota was model group > Meto group > SNT group > normal group, accounting for 15.39%, 10.96%, 7.50% and 1.09% respectively. Bacteria accounting for less than 1% were listed as others. Compared with the normal group, the F/B value decreased in the model group. After treatment with SND and Met, the F/B values both increased. And the adjustment degree of F/B value in the SNT group was better than that in the Meto group (Fig. 11).

### Influence on intestinal mucosa bacterial composition at family level

**Fig. 12:**
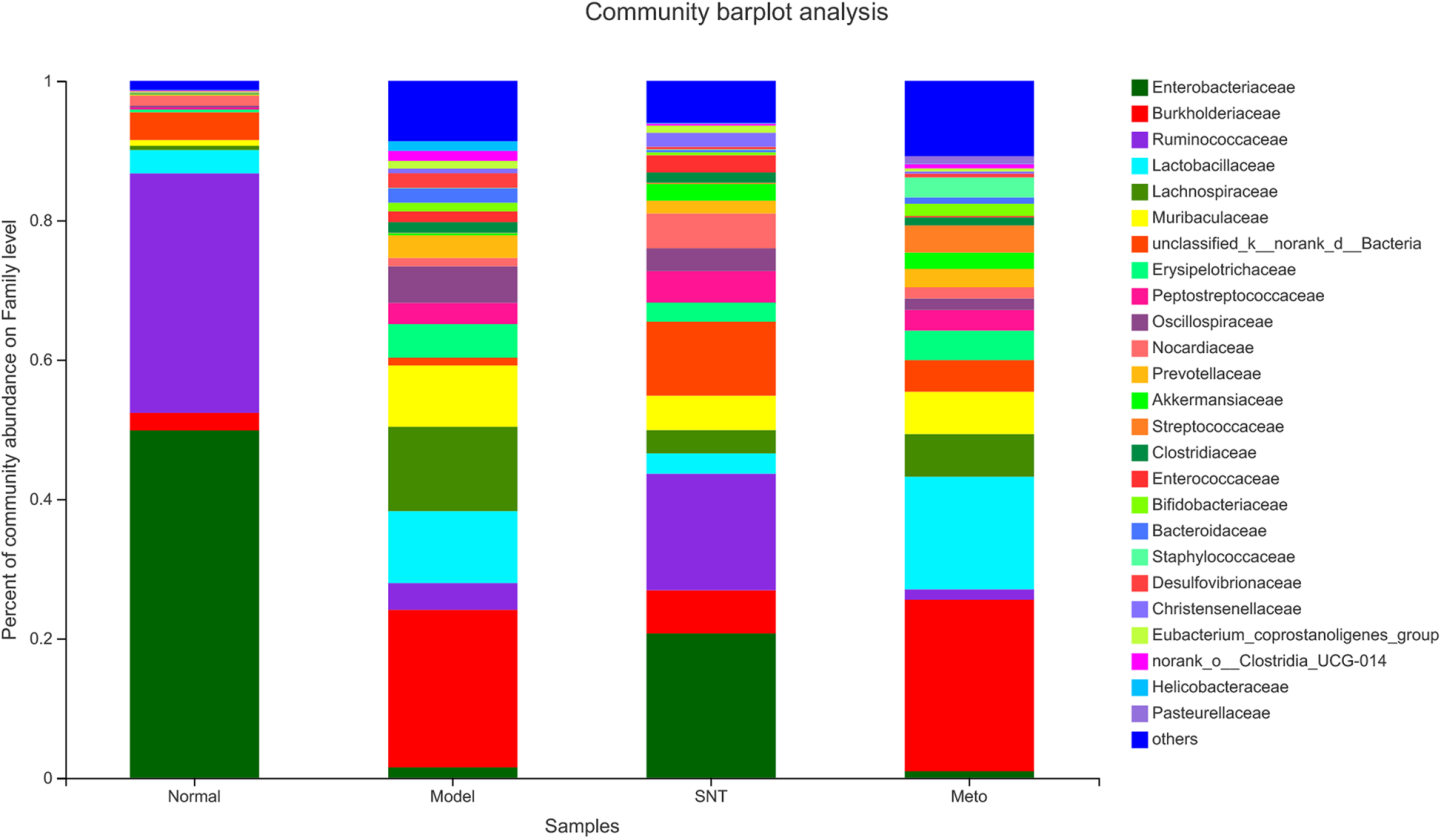
Bacterial community abundance bar plot at the family level. Normal, Model, SNT and Meto represent normal group, model group, Sini Decoction treatment group and metoprolol treatment group, respectively

**Fig. 13:**
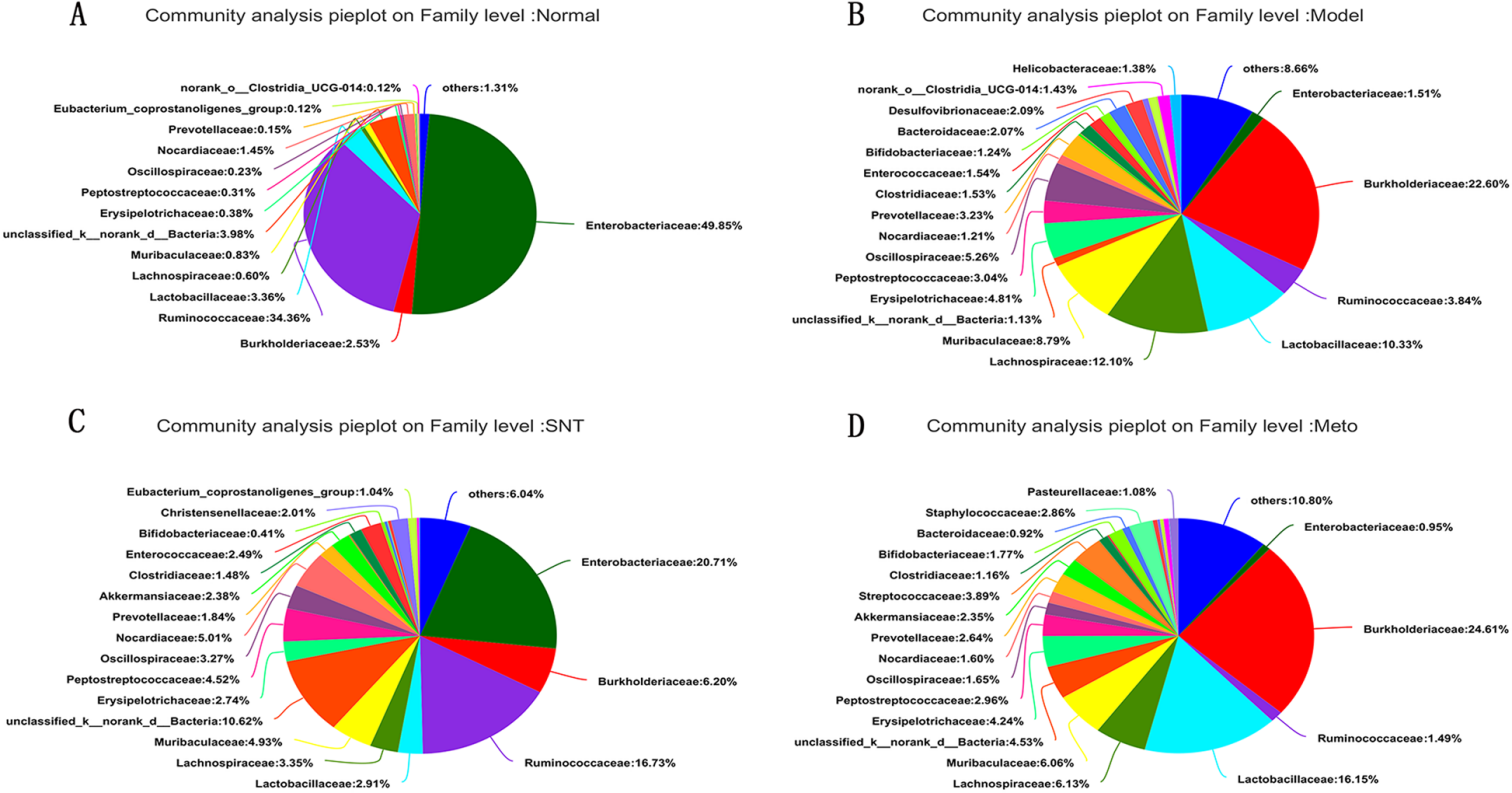
The fan chart reflected the relative abundance of bacteria at the family level. Relative abundance < 1% was classified as others. Normal (**A**), Model (**B**), SNT (**C**), Meto (**D**) represent normal group, model group, Sini Decoction treatment group and metoprolol treatment group, respectively

At the family level, a total of 25 species of bacteria were detected, with relative abundance of more than 1%, and those accounting for less than 1% were listed as Others (Fig. 12). The order of bacteria in the normal group according to relative abundance was Enterobacteriaceae > Ruminococcaceae > unclassified_k__norank_d__Bacteria > Lactobacillaceae. The order of bacteria in the model group according to relative abundance was Burkholderiaceae > Lachnospiraceae > Lactobacillaceae > Muribaculaceae. The order of bacteria in the SNT group according to relative abundance was Enterobacteriaceae > Ruminococcaceae > unclassified_k__norank_d__Bacteria > Burkholderiaceae. The order of bacteria in the Meto group according to relative abundance was Burkholderiaceae > Lactobacillaceae > Lachnospiraceae > Muribaculaceae. The data of relative abundance was shown in the Fig. 13.

**Fig. 14:**
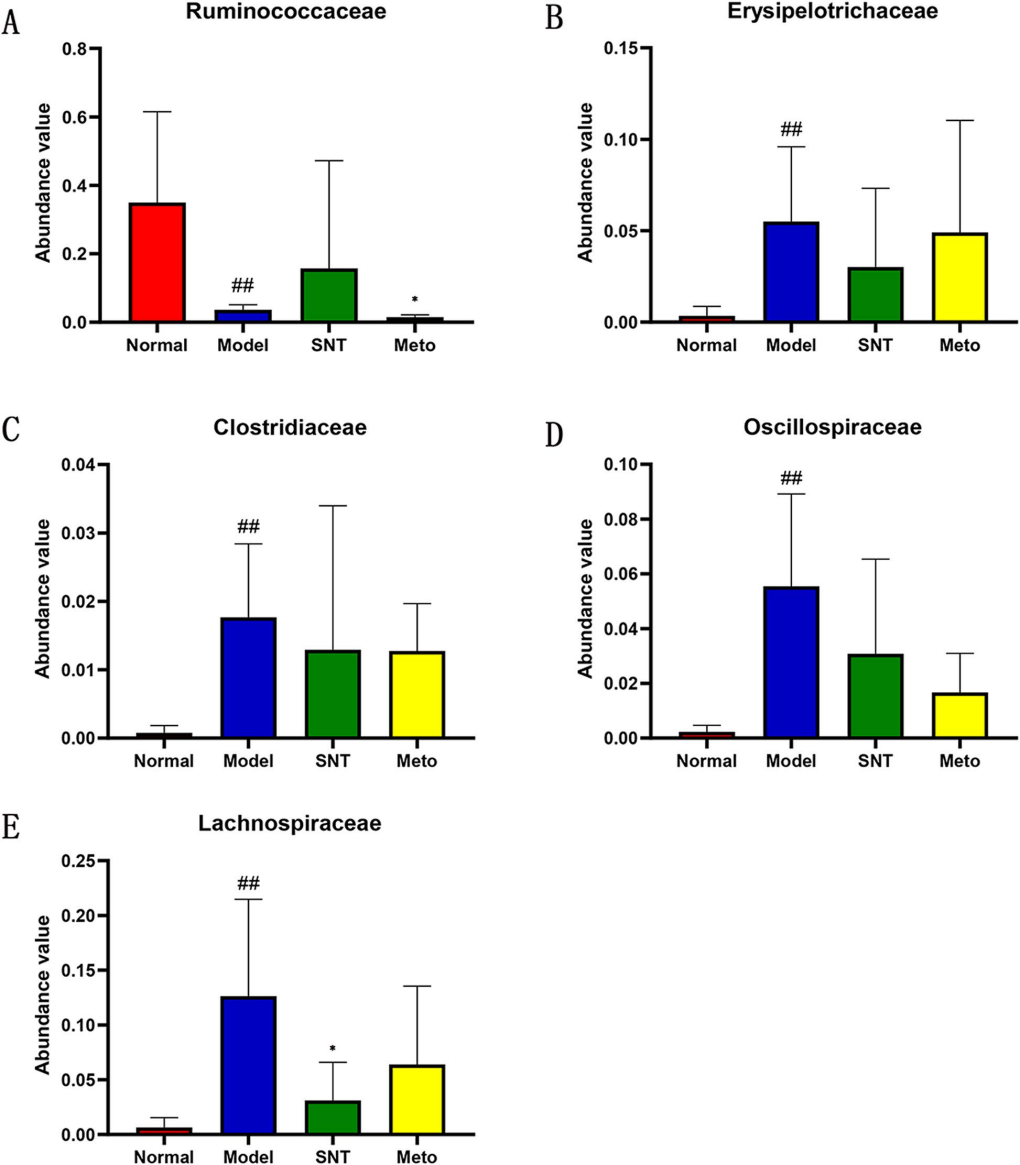
Kruskal–Wallis H test bar plot on bacteria related to HF at the family level. *P < 0.05 versus the model group. ##*P* < 0.01 versus the normal group. Normal, Model, SNT and Meto represent normal group, model group, Sini Decoction treatment group and metoprolol treatment group, respectively

In order to further study the role of intestinal bacteria in the treatment of HF by SND, and to explore the potential mechanism of SND in the treatment of HF, the bacteria closely related to HF were screened out and tested the differences between groups (Fig. 14).

### Linear discriminant analysis (LDA) was used to identify the flora with significant difference in abundance

**Fig. 15:**
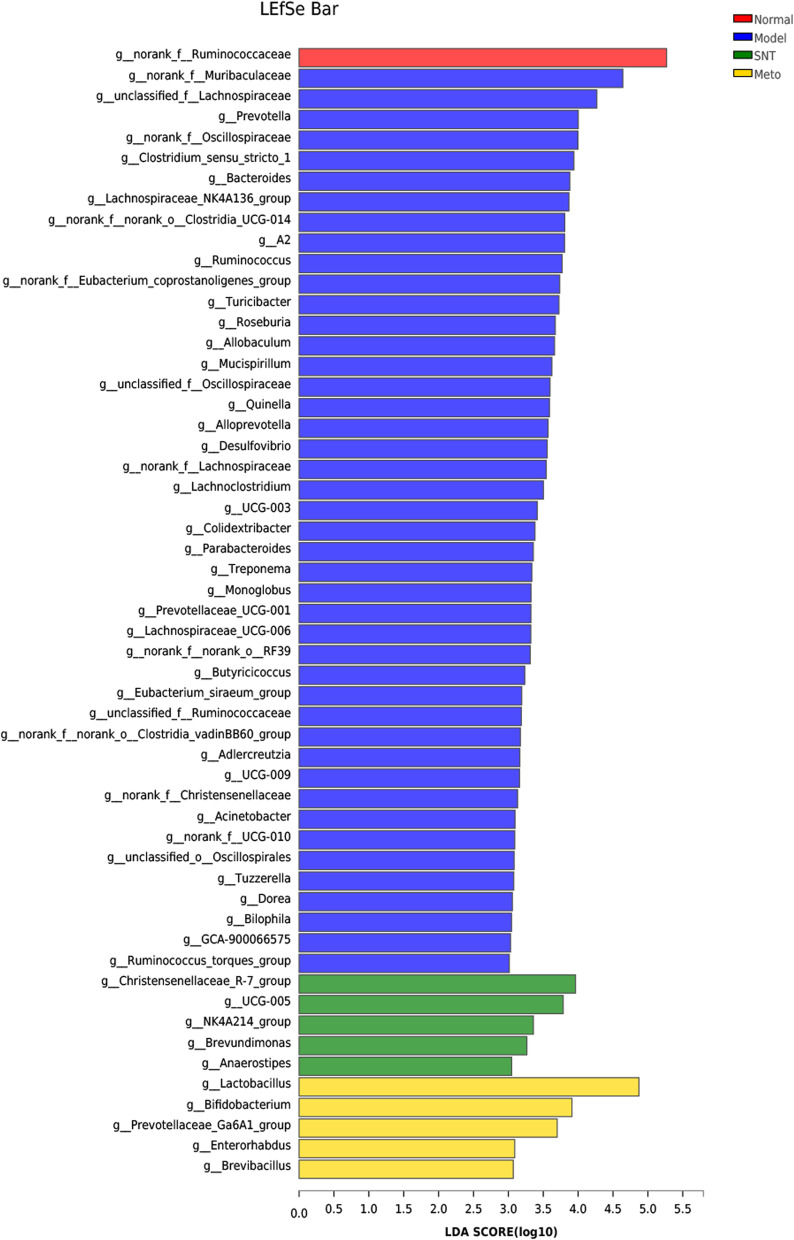
Histogram of the linear discriminant analysis (LDA = 3) value distribution at genus level of intestinal mucosa. Normal, Model, SNT and Meto represent normal group, model group, Sini Decoction treatment group and metoprolol treatment group, respectively

To confirm the bacterial taxonomic differences at genus level among the four groups, LDA was carried out to identify discriminative features (Fig. 15). The main bacteria in the normal group was *norank_f_Ruminococcaceae*; The main bacteria in the model group were *norank_f_Muribaculaceae*, *unclassified_f_Lachnospiraceae*, *Prevotella*, *norank_f_Oscillospiraceae*, etc., mainly opportunistic pathogenic bacteria or harmful bacteria; The characteristic bacteria of the SNT group were *Christensenellaceae_R-7_group*, *UCG-005*, *NK4A214_group*, *Brevundimonas*, *Anaerostipes*; The characteristic bacteria of the Meto group were *Lactobacillus*, *Bifidobacterium*, *Prevotellaceae_Ga6A1_group*, *Enterorhabdus*, *Brevibacillus*. These results indicated that the abundance of harmful bacteria in the intestinal mucosa of HF rats increased, and SND and Met could reduce the abundance of harmful bacteria.

## Discussion

Tang et al. [18, 33, 34] first proposed the "heart failure of intestinal hypothesis". It was pointed out that the blood perfusion of intestinal wall tissue is reduced and the morphology of the intestinal villus membrane is disturbed in HF. Furthermore, due to the increased intestinal permeability caused by the destruction of gut barrier integrity, intestinal bacteria or bacterial secretions are promoted to enter the circulatory system, leading to the further development of inflammation related to HF. As is well-known, HF is related to a chronic inflammatory state [35, 36]. Therefore, it is inferred that intestinal bacteria may not only be regarded as the target of inflammation, but also as a trigger of this pathological mechanism [37].

The echocardiography results showed that compared with normal rats, LVEF and LVFS of the model rats were significantly decreased, suggesting a decrease in cardiac function. After drug treatment, LVEF and LVFS of the rats in the SNT and Meto group were significantly increased compared with the model rats, indicating that the cardiac function of the rats with HF was improved. The same conclusion can be drawn from the H&E staining of myocardial tissue. H&E staining of colon tissue showed that the intestinal wall integrity was damaged in HF rats, suggesting that HF can damage intestinal barrier function and change intestinal homeostasis. The intestinal wall of the SNT group gradually recovered, suggesting that SND could restore the intestinal barrier function. However, the structure of the bowel wall in the Meto group recovered poorly, it is hypothesized that Met had little effect on the gut environment. Studies had shown that the high level of endotoxin LPS in the blood of patients with HF is one of the most effective inducers for the release of pro-inflammatory cytokines. After absorption and distribution into the circulatory system, it can lead to potential inflammation related to HF and has long-term effects [34, 38]. Compared with the normal group, the model group's serum NT-proBNP content and LPS content were significantly increased (*P* < 0.01;*P* < 0.01). Compared with the model group, SND and Met reduced serum NT-proBNP (*P* < 0.01;*P* > 0.05) and LPS (*P* < 0.01;*P* < 0.01) levels. The above results suggest that SND may reduce the level of systemic inflammation by interfering with the intestinal wall structure and thus participate in the process of HF. But the mechanism of Met seems to have nothing to do with repairing the structure of the intestinal wall.

The imbalance of gut flora is closely associated with HF. Studies have confirmed that gut flora plays a key role in the occurrence and progression of HF by mediating several metabolites, including TMAO, SCFAs, and BAs [18]. Experimental results demonstrated that the quantity of OTUs in the model group was higher than the normal group. Similarly, Alpha diversity analysis results indicated that the richness and diversity of intestinal mucosa bacteria in rats with HF increased. However, the higher bacterial richness and diversity does not mean that HF rats have a healthy bacterial flora structure, which may be attributed to the excessive reproduction of harmful bacteria. Previous research had also endorsed this view [39]. After treatment, the number of OTUs in the SNT group decreased. Moreover, the number of OTUs increased further in the Meto group. Alpha diversity analysis results also suggested that SND benefits to restore the bacterial richness and diversity of intestinal mucosa. Met has an insignificant effect of restoring the diversity of the intestinal mucosal flora, but the effect of recovering the richness of the flora is not ideal. Combined with the results of PCoA chart, the distance between the normal group and model group was far. The distance between the SNT and normal group was shortened. And the distance between the Meto and normal group was still far. The above results suggest that SND is significantly better than Met in regulating the bacterial structure of intestinal mucosa in rats with HF.

The differences of bacterial structure among the four groups were analyzed at the phylum level. The experimental results are the same as before. The ratio of Firmicutes to Bacteroidetes (F/B) is an important parameter reflecting intestinal disorders [40]. Compared with the normal group, the F/B value decreased in the model group. After treatment with SND and Met, the F/B values were both adjusted. The results indicate that both SND and Met can play a positive role in intestinal energy metabolism of rats with HF by regulating the abundance of Firmicutes and Bacteroidetes [41].

The family-level histogram analyzed the differences in the microbial flora structure among four groups. Our experiment found that HF is related to the depletion of core gut microbiota, such as Ruminococcaceae. This finding is similar to previous study [37]. The data indicate that changes in the gut microbiota are a potential factor in the onset and progression of HF, as recent findings from other diseases suggest: For example, it has been reported that Lachnospiraceae bacteria are positively correlated with liver disease, kidney disease, metabolic disease, etc. [17]. Similarly, our results could demonstrate that Lachnospiraceae is enriched in rats with HF. In a study on the composition of the intestinal flora of children with obstructive sleep apnoea syndrome, it was observed that the abundance of Clostridiaceae, Oscillospiraceae and Erysipelotrichaceae increased in children with a lower level of mean SaO_2_ during sleep [42]. Hypoxia caused by insufficient breathing is one of the common signs of HF [43]. Our study found that Clostridiaceae, Oscillospiraceae and Erysipelotrichaceae increased significantly in the model group compared with the normal group. The increase in hypoxia-related bacteria can be seen as potential consequences of HF. After treatment with SND, the above-mentioned bacteria Ruminococcaceae, Lachnospiraceae, Clostridiaceae, Oscillospiraceae and Erysipelotrichaceae showed a recovery trend. However, Ruminococcaceae bacteria did not recover in the Meto group. More importantly, Ruminococcaceae is a producer of butyrate [44]. As one of the SCFAs, butyrate plays a positive role in the occurrence and development of HF [45]. It is speculated that the mechanism of SND in the treatment of HF is related to the increase of SCFAs producing bacteria, but the effect of Met in the treatment of HF does not seem to be linked to it.

The results of LDA showed that compared with the normal group, the model group was enriched with 44 kinds of harmful bacteria or conditional pathogens including *norank_f_Muribaculaceae*, *unclassified_f_Lachnospiraceae*, *Prevotella*, *norank_f_Oscillospiraceae*, etc. After treatment with SND and Met, the abundance of these pathogenic bacteria decreased. There are only 5 dominant bacteria in the SNT group, and 5 dominant bacteria in the Meto group. Referring to previous research results [39], it can be believed that the increase in the richness and diversity of intestinal mucosa bacteria in the model group shown by Alpha is due to the excessive reproduction of various harmful bacteria.

## Conclusion

In this study, we observed that the structure and function of the bowel wall of rats with HF were damaged, and the bacterial structure of the intestinal mucosa was obviously changed. These may be not only risk factors for HF, but also potential marks of HF. The bacterial structure of the intestinal mucosa and the intestinal barrier function are restored after treatment, and SND is superior to Met. The findings suggest that SND can affect the development of HF by regulating the intestinal micro-ecological environment. However, gut microbiota composition is fragile in nature, small amount of experimental samples may amplify the possible interference of cage effect. In order to minimize the influence of cage effect as much as possible, we strictly controlled the feeding conditions and replaced the padding material in time. Likewise, individual differences cannot be completely eliminated. Therefore, further large-scale research is needed to prove our findings, which may help to better discover the specific alterations in the intestinal flora of HF.

## Supplementary Information

**Additional file 1:** **Fig S1.** A graphical abstract of the experiment.

**Additional file 2:** **Table S1.** The sequences information of fecal samples. Group: Normal, Model, SNT and Meto represent normal group, model group, Sini Decoction treatment group and metoprolol treatment group, respectively; Seq num: sequences number; Base num: number of bases.

## Data Availability

The datasets generated and/or analysed during the current study are available in the Sequence Read Archive (SRA) repository, BioProject ID: PRJ NA810920, accession numbers: SRP361729. Web link: https://dataview.ncbi.nlm.nih.gov/object/PRJNA810920.

## Abbreviations

HF: Heart failure
CVDs: Cardiovascular diseases
TCM: Traditional Chinese medicine
SND: Sini decoction
SND treatment group: SNT group
Met: Metoprolol
Metoprolol treatment group: Meto group
TMAO: Trimethylamine-N-oxide
SCFAs: Short chain fatty acids
BAs: Bile acids
H&E: Hematoxylin–Eosin
LDA: Linear discriminant analysis
PCoA: Principal coordinate analysis
F/B: Firmicutes to Bacteroidetes ratio
OTUs: Operational taxonomic units
LVEF: Left ventricular ejection fraction
LVFS: Left ventricular fraction shortening
NT-proBNP: N-terminal pro-brain natriuretic peptide
LPS: Lipopolysaccharide
