# Supplementary figures and images for: Bacterial diversity in the intestinal mucosa of heart failure rats treated with Sini Decoction
Source: BMC Complement Med Ther. 2022 Mar 30;22:93. doi: 10.1186/s12906-022-03575-4 (PMC8969309; doi:10.1186/s12906-022-03575-4)

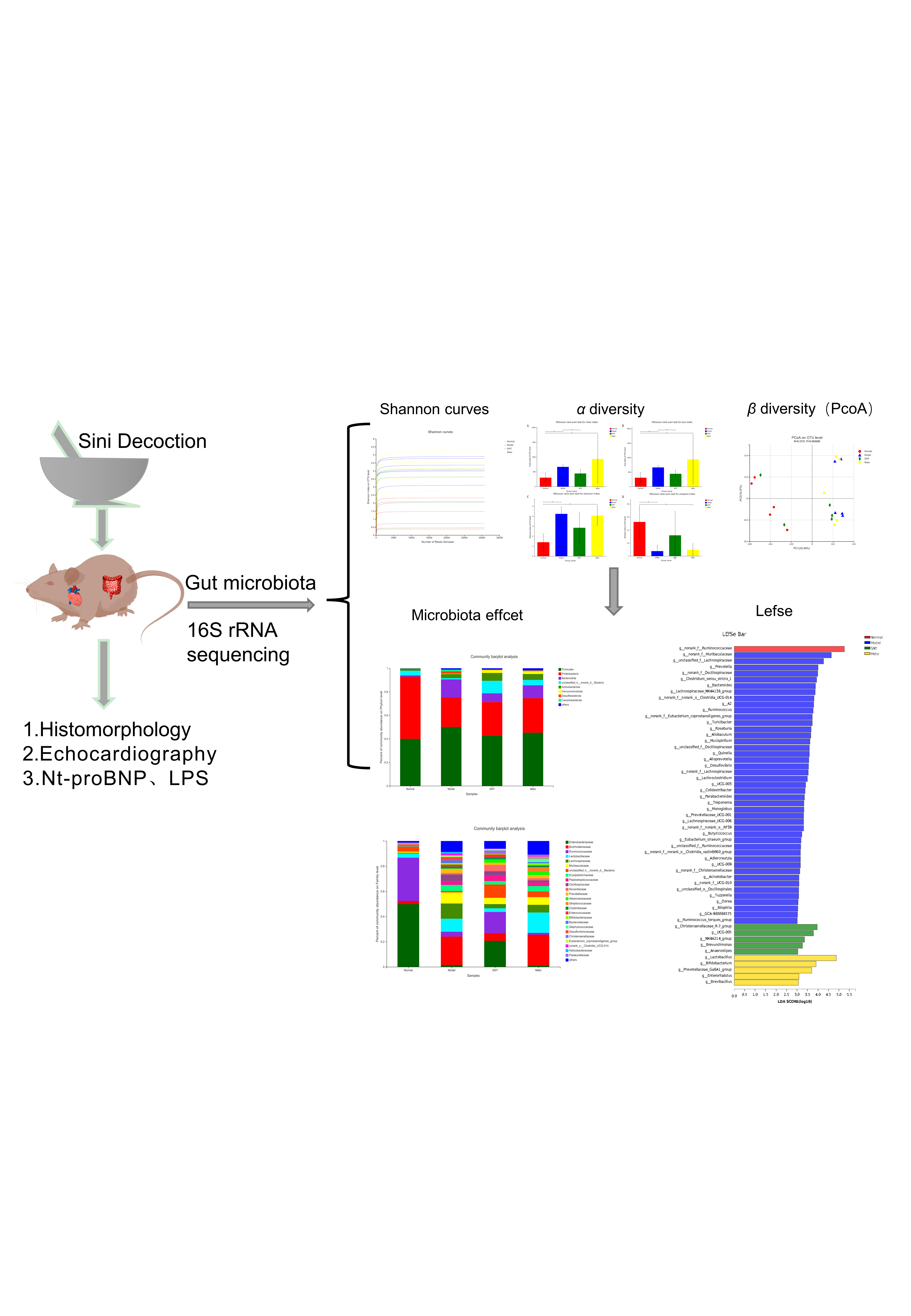

Supplement: Supplementary file 1 — Additional file 1: Fig S1. A graphical abstract of the experiment. [file 12906_2022_3575_MOESM1_ESM.tif]
