# Supplementary material for: Bacterial diversity in the intestinal mucosa of heart failure rats treated with Sini Decoction
Source: BMC Complement Med Ther. 2022 Mar 30;22:93. doi: 10.1186/s12906-022-03575-4 (PMC8969309; doi:10.1186/s12906-022-03575-4)
Supplement: Supplementary file 2 — Additional file 2: Table S1. The sequences information of fecal samples. Group: Normal, Model, SNT and Meto represent normal group, model group, Sini Decoction treatment group and metoprolol treatment group, respectively; Seq num: sequences number; Base num: number of bases. [file 12906_2022_3575_MOESM2_ESM.docx]

| Group | Sample ID | Seq  num | Base  num | Mean  length | Shannon | Simpson | ACE | Chao | Coverage |
| --- | --- | --- | --- | --- | --- | --- | --- | --- | --- |
| Normal | Normal1 | 59883 | 25439736 | 424.82 | 0.7238 | 0.6952 | 182.57 | 185.38 | 0.9990 |
|  | Normal2 | 58374 | 24061794 | 412.20 | 1.4749 | 0.4469 | 542.76 | 480.00 | 0.9961 |
|  | Normal3 | 35399 | 14001658 | 395.54 | 2.0943 | 0.3906 | 314.76 | 322.37 | 0.9987 |
|  | Normal4 | 42079 | 17976486 | 427.21 | 0.3799 | 0.8492 | 84.04 | 90.11 | 0.9994 |
|  | Normal5 | 38988 | 16214363 | 415.88 | 2.3241 | 0.2404 | 413.75 | 434.00 | 0.9976 |
| Model | Model1 | 32035 | 13408593 | 418.56 | 4.0390 | 0.0479 | 555.12 | 550.00 | 0.9977 |
|  | Model2 | 48792 | 20167637 | 413.34 | 4.8928 | 0.0180 | 705.14 | 715.66 | 0.9971 |
|  | Model3 | 40321 | 16603428 | 411.78 | 4.7810 | 0.0165 | 654.98 | 667.08 | 0.9967 |
|  | Model4 | 74610 | 31398189 | 420.83 | 4.3647 | 0.0582 | 762.04 | 762.12 | 0.9963 |
|  | Model5 | 52868 | 22392067 | 423.55 | 3.1073 | 0.2385 | 635.52 | 639.14 | 0.9969 |
| SNT | SNT1 | 47877 | 19504207 | 407.38 | 2.0413 | 0.5163 | 477.99 | 509.33 | 0.9980 |
|  | SNT2 | 37990 | 15636348 | 411.59 | 3.6272 | 0.0900 | 402.62 | 414.81 | 0.9985 |
|  | SNT3 | 41512 | 17772129 | 428.12 | 0.4546 | 0.8803 | 239.50 | 214.73 | 0.9981 |
|  | SNT4 | 52781 | 21921973 | 415.34 | 3.9857 | 0.0409 | 522.75 | 512.80 | 0.9974 |
|  | SNT5 | 39532 | 14490211 | 366.54 | 4.1159 | 0.0656 | 567.84 | 568.12 | 0.9980 |
| Meto | Meto1 | 65410 | 27856603 | 425.88 | 2.7416 | 0.2524 | 537.15 | 542.08 | 0.9969 |
|  | Meto2 | 56465 | 24009167 | 425.20 | 3.5762 | 0.1146 | 649.95 | 679.18 | 0.9967 |
|  | Meto3 | 38077 | 15094107 | 396.41 | 4.5525 | 0.0267 | 588.09 | 585.52 | 0.9982 |
|  | Meto4 | 61856 | 25886574 | 418.50 | 5.3169 | 0.0454 | 2461.94 | 2396.44 | 0.9872 |
|  | Meto5 | 46134 | 19047728 | 412.88 | 4.1598 | 0.0411 | 470.36 | 472.81 | 0.9982 |

Table1: The sequences information of fecal samples
